# Greenhouse Gas Emissions from Soils Amended with Cornstalk Biochar at Different Addition Ratios

**DOI:** 10.3390/ijerph20020927

**Published:** 2023-01-04

**Authors:** Yongchun Zhou, Danyang Li, Zhenglong Li, Sibo Guo, Zhimin Chen, Liulin Wu, Yan Zhao

**Affiliations:** 1 School of Resources and Civil Engineering, Northeastern University, No. 3–11, Wenhua Road, Heping District, Shenyang 110819, China; 2 Institute for Frontier Technologies of Low-Carbon Steelmaking, Northeastern University, No. 3–11, Wenhua Road, Heping District, Shenyang 110819, China

**Keywords:** CO_2_ emission, N_2_O emission, CH_4_ emission, global warming potential, priming effect, native soil organic carbon mineralization

## Abstract

Biochar addition has been recommended as a potential strategy for mitigating climate change. However, the number of studies simultaneously investigating the effects of biochar addition on CO_2_, N_2_O and CH_4_ emissions and sequentially global warming potential (GWP) is limited, especially concerning its effect on native soil organic carbon (SOC) mineralization. An incubation experiment was conducted to investigate soil physicochemical properties, CO_2_, N_2_O and CH_4_ emissions and GWP in the treatments with 0% (CK), 1% (BC1) and 4% (BC4) cornstalk biochar additions, and clarify the priming effect of biochar on native SOC mineralization by the ^13^C tracer technique. Generally, biochar addition increased soil pH, cation exchange capacity, SOC and total nitrogen, but decreased NH_4_^+^-N and NO_3_^−^-N. Compared with CK, BC1 and BC4 significantly reduced CO_2_ emissions by 20.7% and 28.0%, and reduced N_2_O emissions by 25.6% and 95.4%, respectively. However, BC1 significantly reduced CH_4_ emission by 43.6%, and BC4 increased CH_4_ emission by 19.3%. BC1 and BC4 significantly reduced the GWP by 20.8% and 29.3%, but there was no significant difference between them. Biochar addition had a negative priming effect on native SOC mineralization, which was the reason for the CO_2_ emission reduction. The negative priming effect of biochar was attributed to the physical protection of native SOC by promoting microaggregate formation and preferentially using soluble organic carbon in biochar. The N_2_O emission decrease was rooted in the reduction of nitrification and denitrification substrates by promoting the microbial assimilation of inorganic nitrogen. The inconsistency of CH_4_ emissions was attributed to the different relative contributions of CH_4_ production and oxidation under different biochar addition ratios. Our study suggests that 1% should be a more reasonable biochar addition ratio for mitigating greenhouse gas emissions in sandy loam, and emphasizes that it is necessary to furtherly investigate nitrogen primary transformation rates and the relative contributions of CH_4_ production and oxidation by the ^15^N and ^13^C technique, which is helpful for comprehensively understanding the effect mechanisms of biochar addition on greenhouse gas emissions.

## 1. Introduction

Global climate change characterized by global warming is threatening the balance of natural ecosystems and human survival and development [1]. It has been stated that climate change will be observed everywhere, and temperatures will continue to rise over the next three decades [2]. Therefore, how to deal with global climate change has become a serious challenge for mankind, and reducing greenhouse gas emissions is the key to mitigating global warming [3]. Agricultural soil is one of the most important sources of greenhouse gas emissions and is estimated to account for 13.5% of total global greenhouse gas emissions [4]. Therefore, agricultural soil plays an important role in reducing greenhouse gas emissions, increasing the carbon sink effect and mitigating climate change.

Biochar is a solid material produced by the pyrolysis of biomass under completely or partially anaerobic conditions at high temperatures. Biochar addition is considered to be a promising measure for reducing greenhouse gas emissions and increasing the effectiveness of soil carbon sinks [5]. On the one hand, biochar is mainly composed of anti-biodegradable aromatic compounds, which are inert and have a slow decomposition rate. Thus, biochar can be stable in soil and improve soil stable carbon storage [5,6]. On the other hand, biochar is generally characterized by a large surface area, abundant micropores and a strong cation exchange capacity, which affect soil physicochemical and biological characteristics, and then affect soil carbon and nitrogen dynamics, and ultimately affect soil greenhouse gas emissions [7,8]. The effects of biochar addition on soil greenhouse gas emissions have been extensively studied, but the results are inconsistent. For CO_2_, some studies have found that biochar addition promoted soil CO_2_ emissions [9,10,11,12]. For example, Mitchell et al. [9] found that sugar maple biochar addition increased CO_2_ emissions, and the CO_2_ emissions were largest at the lowest addition amount of 5t ha^−1^. Meanwhile, Tang et al. [10] found that CO_2_ emissions increased with the increasing biochar addition ratio. However, some investigations contradict the conclusions shown above [13,14,15,16]. For example, Sakin et al. [16] found that biochar addition reduced soil CO_2_ emissions, and the CO_2_ emissions were lowest at the highest addition ratio. It has also been found that biochar addition reduced CO_2_ emissions in forest soil but had no significant effect on CO_2_ emissions in grassland soil [7]. For N_2_O, most studies have found that biochar addition inhibited soil N_2_O emissions [17,18,19,20]. However, some studies have found that the biochar addition promoted N_2_O emissions [21,22]. It has also been found that 550 °C pine chip biochar significantly increased soil N_2_O emissions, but 900 °C walnut shell biochar had no significant effect on N_2_O emissions [21]. For CH_4_, because paddy soil is the main source of CH_4_ emissions, most previous studies on CH_4_ emissions were conducted in paddy soil, and it has mostly been found that biochar addition inhibited soil CH_4_ emissions [23,24,25]. However, some studies have also found that biochar addition promoted CH_4_ emissions [26,27].

The inconsistency in greenhouse gas emissions may be rooted in the different biochar materials, pyrolysis temperatures, addition ratios and soil types. Therefore, further research is needed. Moreover, although CH_4_ emissions are lower in soil types other than paddy soil, the contribution of CH_4_ to the greenhouse effect is still significant due to its higher global warming potential (GWP), which is 25 times that of CO_2_ [28]. Therefore, it is essential to investigate the effect of biochar addition on CH_4_ emission in other soil types. At the same time, biochar addition may impose different effects on the emission of CO_2_, N_2_O and CH_4_, and the GWPs of these gases were different, thus, the simultaneous measurement of the three greenhouse gases and the subsequent calculation of the GWP are necessary to accurately assess the effect of biochar addition on mitigating global warming, but there are relatively few studies that simultaneously measure the three greenhouse gases. More importantly, although the effect of biochar addition on soil CO_2_ emissions was mostly attributed to the priming effect of biochar addition on native soil organic carbon (SOC) mineralization, few studies investigated the priming effect directly. The carbon stable isotope technique has outstanding advantages in carbon dynamic research. The ^13^C tracer technique can trace the source of CO_2_, quantitatively analyze the proportion of CO_2_ emissions from soil and biochar and then clarify whether the priming effect is positive or negative. For example, Lu et al. [29] found that cornstalk biochar addition had a negative priming effect using the ^13^C tracer technique. However, they did not consider the priming effect of different biochar addition ratios on native SOC decomposition. To fill these knowledge gaps, the main objectives of the present study were (i) to investigate the effects of biochar addition at different addition ratios on the fluxes and cumulative emissions of CO_2_, N_2_O and CH_4_ and the GWP; (ii) to clarify the priming effect of biochar addition at different addition ratios on native SOC decomposition by the ^13^C tracer technique. It is hypothesized that: (i) biochar addition may promote soil CO_2_ emissions in the early stage, due to the decomposition of the soluble organic carbon contained in biochar; (ii) the GWP may decrease with increasing biochar addition ratios, because biochar addition would inhibit CO_2_, N_2_O and CH_4_ emissions by influencing carbon and nitrogen dynamics; (iii) biochar addition would have a negative priming effect on the decomposition of native SOC.

## 2. Materials and Methods

### 2.1. Soil Sampling and Biochar

The experimental soil was collected in March 2021 from the surface soil (0–20 cm) of the experimental field in the Liaoning Academy of Agricultural Sciences. The soil texture is sandy loam. The soil was air-dried and stripped of stones and the miscellaneous roots of plants and then passed through a 2 mm sieve for use. The experimental biochar was made from the pyrolysis of cornstalk at 400 °C, which was purchased from Henan Zhongxin Blue Sky Environmental Protection Equipment Co., Ltd. The physicochemical characteristics of soil and biochar are shown in Table 1.

### 2.2. Design of the Incubation Experiment

The experiment consisted of three levels of biochar treatments, no-biochar (CK), 1% biochar added (BC1), 4% biochar added (BC4), with 12 replicates for each treatment, three replicates were used for gas collection and final destructive soil sampling, and nine replicates were used for three destructive soil samplings during incubation. All experimental soils were conditioned to 40% of the field capacity before the incubation experiments, and the soils were pre-incubated for 7 days to activate and stabilize the microorganisms. Thirty-six conical flasks (250 mL) were prepared with 50 g (oven-dry basis) soil, then biochar was added and mixed well with soil at ratios of 0% (CK), 1% (BC1) and 4% (BC4) (w/w). Inorganic nitrogen (NH_4_)_2_SO_4_ was added to all treatments by solution at an addition rate of 100 mg N kg^−1^, and then the soil water content was adjusted to 60% of the field water capacity with deionized water. In order to keep the soil water content, deionized water was added by weighing the conical flask every day during the incubation process. The pre-incubation and subsequent incubation were conducted under the conditions of constant temperature (25 ± 1 °C) and darkness. All conical flasks were covered with pinhole parafilm to maintain aerobic conditions and reduce water loss. The gas collections were performed on the 1st, 3rd, 5th, 7th, 9th, 13th and 30th days of the incubation period to measure the concentrations of CO_2_, N_2_O and CH_4_, while the δ^13^C values of CO_2_ were measured on the 1st, 3rd, 9th, 13th and 30th days. Before gas collection, the sealing film of the conical flask was replaced by a rubber plug with a three-way valve. The inlet and outlet of the three-way valve were connected to the flask and the direct rotary vane vacuum pump (2XZ type), respectively. The vacuum was pumped by the direct rotary vane vacuum pump for 35 s and then flushed with ambient air, and this operation was repeated three times. At the same time, the ambient air was collected as a zero-time sample for analysis. Then, the conical flask was sealed and put back into the incubator for 4 h. After that, 40 mL of top air in the flask was sampled with a syringe and then injected into two 20 mL pre-vacuumed air bags, respectively. One was used to measure the gas concentration, and another was used to measure the δ^13^C of CO_2_. The gas samples were stored in a −20 °C refrigerator and determined as soon as possible. Destructive soil samplings were conducted to measure soil pH, SOC, TN, NH_4_^+^-N, NO_3_^−^-N and CEC on the 1st, 3rd and 13th days, and destructive soil sampling was also carried out on the 30th day to determine the above soil physicochemical properties after gas collection.

### 2.3. Samples Determination

Soil/water (1:2.5) and biochar/water (1:20) suspensions were configured to measure the pH of the soil and biochar using a pH meter (PHS-25, Shanghai, China). Fresh soil samples were extracted with 2 mol/L KCl, and the concentrations of NH_4_^+^-N and NO_3_^−^-N were determined by an ultraviolet spectrophotometer (DR 6000, Loveland, CO, USA). SOC was determined by the potassium dichromate external heating method and TN was determined by the Kjeldahl nitrogen method [30]. CEC was determined by the ammonium acetate method [30]. Gas concentrations were determined by a gas chromatograph (Agilent 7890, Santa Clara, CA, USA) with a flame ionization detector (FID) at 250 °C for CO_2_ and CH_4_ and an electron capture detector (ECD) at 360 °C for N_2_O. The soil samples and biochar samples were finely ground with a ball mill. Before the measurement of the soil sample isotopes, the soil powder was treated with 0.1 mol/L HCl solution for 24 h to remove carbonates, then washed with deionized water to remove HCl, and finally dried at 65 °C to a constant weight. The carbon isotope composition (δ^13^C) of the soil and biochar samples was determined by an elemental analyzer (FlashEA2000) coupled with an isotope mass spectrometer (MAT253, Bremen, Germany). The δ^13^C of CO_2_ was determined by an isotope mass spectrometer with a trace gas preconcentration device. The δ^13^C value of the sample was calculated as follows:

(1) $$\delta^{13}C\left(‰\right)=\left[\frac{R_{\mathrm{samples}}-R_{\mathrm{standard}}}{R_{\mathrm{standard}}}\right]\times 1000$$

where $R_{\mathrm{samples}}$ and $R_{\mathrm{standard}}$ are the ratios of heavy elements to light elements in the samples and standards, respectively, and the solid standard is VPDB, and the gas standard is a fixed-value CO_2_ standard gas and the measurement accuracy of the isotopic composition is 0.1‰.

### 2.4. Calculations

Soil greenhouse gas fluxes (CO_2_, mg C kg^−1^ h^−1^; N_2_O and CH_4_, mg kg^−1^ h^−1^) were calculated using the following formula [31]:

(2) $$F_{\mathrm{total}}=\left[\left(C_{2}-C_{1}\right)\times M\times V_{g}\times 10^{3}\right]/\left(W\times {\mathrm{MV}}_{\mathrm{corr}}\times t\right)$$

where C_1_ and C_2_ are soil greenhouse gas concentrations (μL L^−1^) at 0 time of gas collection and after 4 h of culture flask closure, respectively. M is the molecular weight of CO_2_-C, N_2_O and CH_4_ (g mol^−1^); V_g_ is the volume of the culture flask (m^3^); W is soil quality (kg); t is culture bottle closing time (4 h); MV_corr_ is the molecular volume adjusted by temperature (m^3^ mol^−1^), which is corrected by the following formula [32]:

(3) $${\mathrm{MV}}_{\mathrm{corr}}=0.02241\times \frac{273.15+T}{273.15}$$

where T is the indoor temperature (25 °C), 0.02241 (m^3^) is the ideal molar volume of gas at 273.15 K and 1 standard atmospheric pressure.

Soil cumulative greenhouse gas emissions during incubation ($T_{{\mathrm{CO}}_{2}}$, mg C kg^−1^ h^−1^; $T_{N_{2}O}$ and $T_{{\mathrm{CH}}_{4}}$, mg kg^−1^ h^−1^) were calculated by the following formula [33]:

(4) $$T=\overset{n}{\underset{i=1}{\sum }}\frac{F_{i}+F_{i+1}}{2}\times \left(t_{i+1}-t_{i}\right)$$

where F_i_ and F_i+1_ are the fluxes of soil greenhouse gas (CO_2_, N_2_O and CH_4_ (μL L^−1^)) collected at i and i + 1, respectively, t_i+1_ − t_i_ is the time interval between the two gas collections and n is the total number of gas collections.

The CO_2_ flux from SOC decomposition (F_soil_, mg C kg^−1^ h^−1^) was calculated using the following formula [34]:

(5) $$F_{\mathrm{soil}}=\left(1-f_{b}\right)\times F_{\mathrm{total}}$$

where F_total_ is the total CO_2_ flux from biochar-added soil; f_b_ is the ratio of CO_2_ flux from biochar to the total CO_2_ flux calculated by the following formula [35]:

(6) $$f_{b}=\frac{\delta -\delta_{s}}{\delta_{b}-\delta_{s}}$$

where $\delta_{s}$ is the δ^13^C value of native SOC; $\delta_{b}$ is the δ^13^C value of organic carbon in biochar; δ is the δ^13^C value of CO_2_ released from biochar-added soil, which is corrected by the following formula [34]:

(7) $$\delta =\left(\delta_{2}\times C_{2}-\delta_{1}\times C_{1}\right)/\left(C_{2}-C_{1}\right)$$

where $\delta_{1}$ and $\delta_{2}$ are δ^13^C values of CO_2_ at 0 time of gas collection and 4 h after the closure of the culture flask.

The priming effect of biochar (Δ) was calculated by the following formula [34]:

(8) $$\Delta =F_{\mathrm{soil}}-F_{\mathrm{soil}-\mathrm{ck}}$$

where F_soil_ is the CO_2_ flux generated by SOC decomposition with biochar addition; F_soil-ck_ is the CO_2_ flux of the control (without biochar addition) treatment.

The GWP was calculated by the following formula [36]:

(9) $$\mathrm{GWP}=44/12T_{{\mathrm{CO}}_{2}}+298T_{N_{2}O}+25T_{{\mathrm{CH}}_{4}}$$

where $T_{{\mathrm{CO}}_{2}}$, $T_{N_{2}O}$ and $T_{{\mathrm{CH}}_{4}}$ are the 30-day cumulative emissions of CO_2_, N_2_O and CH_4_ ($T_{{\mathrm{CO}}_{2}}$, mg C kg^−1^ h^−1^; $T_{N_{2}O}$ and $T_{{\mathrm{CH}}_{4}}$, mg kg^−1^ h^−1^). The default molecular weights of CH_4_ and N_2_O in 100 years were 25 and 298, respectively, and the GWP value of CO_2_ was 1.

### 2.5. Data Analyses

All data were analyzed by SPSS (version 26.0, SPSS Inc., Chicago, CA, USA) and mapped by Origin (version 9.0, OriginLab, Northampton, MA, USA). The differences among different treatments were analyzed using one-way ANOVA (α = 0.05).

## 3. Results

### 3.1. The Effects of Biochar Addition on Soil Characteristic

**Figure 1:**
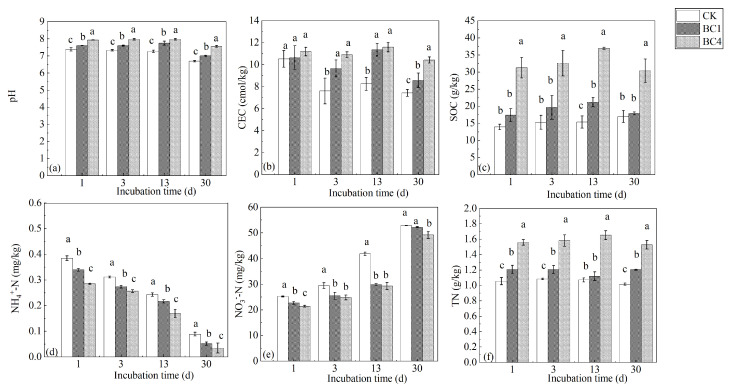
The effects of biochar addition on soil (**a**) pH, (**b**) CEC, (**c**) SOC, (**d**) NH_4_^+^-N, (**e**) NO_3_^−^-N and (**f**) TN during the 30-day incubation. Different letters indicate significant variations among treatments at the same incubation time at *p* < 0.05. Vertical bars indicate the standard error of the mean (n = 3).

Biochar addition increased the soil pH, and BC4 > BC1 > CK (Figure 1a). At the beginning of the incubation period, there was no significant difference in CEC among CK, BC1 and BC4. As the incubation time increased, the CEC of BC1 and BC4 were significantly higher than that of CK. While there was no significant difference between BC1 and BC4 on the 3rd and 13th days, in the later stage of incubation, the CEC of BC1 and BC4 were significantly higher than that of CK, and that of BC4 was significantly higher than that of BC1 (Figure 1b). Biochar addition increased SOC, and BC4 was significantly higher than CK (Figure 1c). Compared with CK, the concentrations of NH_4_^+^-N and NO_3_^−^-N in biochar treatments (BC1 and BC4) decreased by 26.2%–61.1% and 1.19%–28.7%, respectively, but the TN content increased by 3.7%–54.4% (Figure 1d–f). With the extension of incubation time, NH_4_^+^-N content showed a trend of gradual decline, while NO_3_^−^-N content increased (Figure 1d,e).

### 3.2. The Effects of Biochar Addition on Soil CO_2_, N_2_O and CH_4_ Emissions

**Figure 2:**
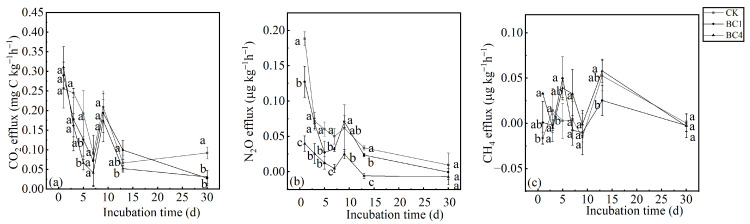
The effects of biochar addition on soil (**a**) CO_2_, (**b**) N_2_O and (**c**) CH_4_ effluxes during the 30-day incubation. Different letters indicate significant variations among treatments at the same incubation time at *p* < 0.05. Vertical bars indicate the standard error of the mean (n = 3).

At the beginning of the incubation period, soil CO_2_ effluxes of biochar treatments (BC1 and BC4) were higher than CK, but the differences were not significant. As the incubation time increased, the soil CO_2_ effluxes of BC1 and BC4 treatments were lower than those of CK, and BC1 and BC4 treatments were significantly different from CK on the 30th day, while there was no significant difference between BC1 and BC4 (Figure 2a). The soil CO_2_ efflux of the BC1, BC4 and CK treatments showed a similar change pattern with the incubation time, i.e., soil CO_2_ efflux decreased rapidly with the incubation time before the 7th day, but increased on the 9th day, and then continued to decline. It should be noted that the CO_2_ efflux of CK increased slightly in the later stage of incubation time (Figure 2a).

The soil N_2_O efflux reached the maximum on the first day, and then declined rapidly, but increased slightly on the 9th day, and finally declined to a stable level (Figure 2b). Except for on the 9th day, soil N_2_O efflux was BC4 < BC1 < CK, and the difference was significant among CK, BC1 and BC4 on the 1st, 7th, and 13th days. However, there was no significant difference among CK, BC1 and BC4 on the 30th day (Figure 2b).

The CH_4_ effluxes of the three treatments had no obvious pattern, and their values fluctuated between −0.25 μg kg^−1^ h^−1^ and 0.08 μg kg^−1^ h^−1^, and it should be noted that there was no significant difference among the three treatments on the 30th day (Figure 2c).

**Figure 3:**
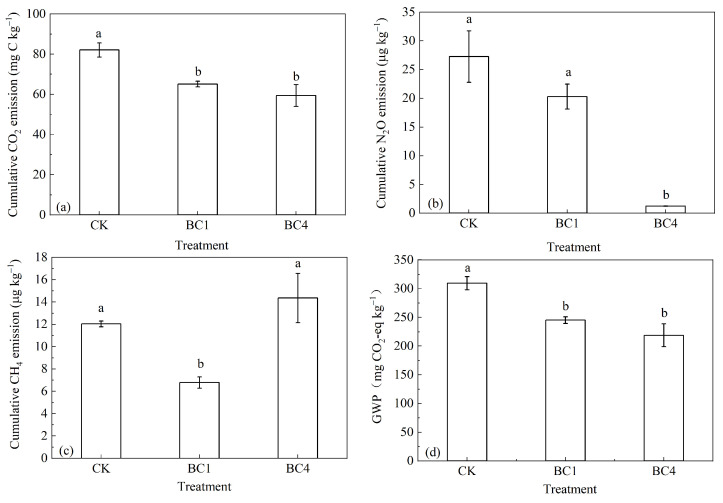
The effects of biochar addition on soil cumulative (**a**) CO_2_, (**b**) N_2_O and (**c**) CH_4_ emissions and (**d**) GWP during the 30-day incubation. Different letters indicate significant variations among treatments at the same incubation time at *p* < 0.05. Vertical bars indicate the standard error of the mean (n = 3).

Compared with CK, BC1 and BC4 significantly reduced the cumulative CO_2_ emissions by 20.7% and 28.0% during the whole incubation, while there was no significant difference between BC1 and BC4 (Figure 3a). Compared with CK, BC1 and BC4 reduced the cumulative N_2_O emissions by 25.6% and 95.4%, while the difference between BC1 and CK was not significant. It should be noted that BC4 treatment was significantly lower than CK and BC1 (Figure 3b). Compared with CK, BC1 significantly reduced CH_4_ emissions by 43.6%, and BC4 increased CH_4_ emissions by 19.3% and the difference between BC4 and CK was not significant (Figure 3c). Compared with CK, BC1 and BC4 significantly reduced the GWP by 20.8% and 29.3%; the GWP of BC4 was 10.7% lower than that of BC1, but there was no significant difference between them (Figure 3d).

### 3.3. The Priming Effects of Biochar Addition on Soil Organic Carbon Mineralization

**Figure 4:**
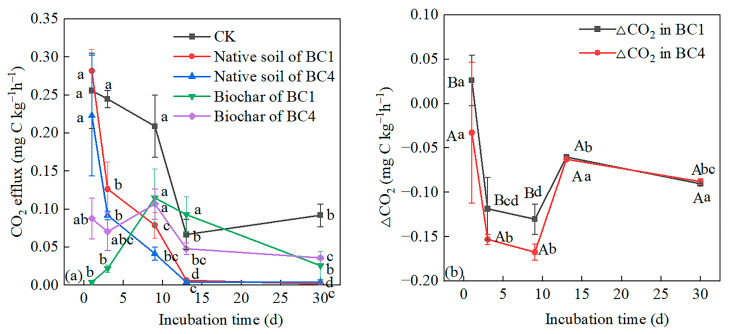
CO_2_ efflux derived from the (**a**) native soil, biochar and (**b**) ∆CO_2_ in soils added with 1% and 4% biochar additions during the 30-day incubation. ∆CO_2_ is the difference in CO_2_ efflux from native soil between biochar addition treatment and CK, i.e., the priming effect of biochar. Different capital letters indicate significant differences among different treatments at the same incubation period, and different lowercase letters indicate significant differences among different incubation time at the same treatments (*p* < 0.05). Vertical bars indicate the standard error of the mean (n = 3).

Except for on the 1st day, the native soil CO_2_ fluxes of BC1 were lower than those of CK, and the native soil CO_2_ fluxes of BC4 were lower than those of CK throughout the incubation process. As the incubation time increased, BC1 and BC4 treatments showed a similar change pattern, i.e., native soil CO_2_ fluxes decreased rapidly before the 13th day and then tended to a stable level (Figure 4a). With the extension of incubation time, the biochar CO_2_ fluxes of the treatments (BC1 and BC4) reached the maximum on the 9th day and then decreased (Figure 4a). For BC1, ∆CO_2_ was positive on the first day, indicating a positive priming effect of biochar on native SOC; ∆CO_2_ was negative in the later stage, which indicated a negative priming effect. For BC4, ∆CO_2_ was negative throughout the incubation process, indicating a negative priming effect (Figure 4b). The negative priming effects of BC1 and BC4 reached their maximum value on the 9th day and then decreased significantly on the 13th day, and gradually stabilized in the later stage (Figure 4b). The negative priming effect of BC4 was significantly stronger than that of BC1 before the 9th day, while there was no significant difference between them in the later stage (Figure 4b).

## 4. Discussion

### 4.1. Effects of Biochar Addition on Soil Characteristic

Biochar addition significantly increased soil pH; this result is consistent with the previous studies [37,38]. One possibility is that biochar contains soluble ash elements K, Ca and Mg, and these alkaline ions increased soil pH [38]. Another possibility is that the oxygen-active groups -COOH and -OH present on the biochar surface reacted with metal cations in the soil to form metal ion complexes, resulting in an increase in soil pH [37]. Most previous studies showed that biochar addition increased soil CEC [39,40]. The increasing CEC should be attributed to the high CEC (13.25 cmol kg^−1^) of biochar; a higher biochar CEC level may be due to its high specific surface area and porosity [39]. Many studies have verified that biochar addition increases SOC [41,42]. The ability of biochar addition to improve SOC may be due to the high carbon content of biochar [5]. In addition, biochar addition can adsorb and then inhibit the mineralization of active organic matter, gradually increasing the stability of carbon, and thus improving SOC [41]. It should be noted that we could not determine whether the native SOC increases or decreases with biochar addition, which is one of the purposes of our research and will be further discussed later. With the extension of incubation time, the concentration of NH_4_^+^-N decreased while the concentration of NO_3_^−^-N increased, indicating that the nitrification reaction was undergoing in the incubation process, and NH_4_^+^-N transformed into NO_3_^−^-N. Biochar addition led to a decrease in NH_4_^+^-N and NO_3_^−^-N concentrations, but an increase in the TN concentration, which may be because the biochar addition promoted the assimilation of NH_4_^+^-N and NO_3_^−^-N by microorganisms. Previous studies have shown that microorganisms may assimilate more inorganic nitrogen in soils with high organic matter content and high C/N content [43,44]. Biochar addition increased SOC content (Figure 1c), which should provide more carbon sources and then improve the microbial assimilation of NH_4_^+^-N and NO_3_^−^-N. However, we did not investigate the effect of biochar addition on microbial biomass nitrogen, which should be carried out in the future to verify the above mechanism.

### 4.2. Effects of Biochar Addition on Soil CO_2_, N_2_O and CH_4_ Emissions

The CO_2_ efflux of biochar addition treatments was higher than that of CK at the beginning of the incubation period, which verified our first hypothesis. Other studies also found a similar increase in the CO_2_ efflux after biochar addition [11,12]. The ability of biochar addition to promote soil CO_2_ efflux was attributed to the degradation of soluble organic carbon in biochar itself [38] and the positive priming effect of biochar addition on native SOC mineralization [45]. For BC1, the positive priming effect played a dominant role in increasing CO_2_ emissions at the beginning of the incubation period (Figure 4b), because the CO_2_ efflux from biochar was very low and near zero on the 1st day (Figure 2a). For BC4, although there was a negative priming effect (Figure 4b), the CO_2_ efflux from biochar was high due to more biochar containing more soluble organic carbon on the 1st day (Figure 2a). However, it should be noted that although the CO_2_ efflux from biochar was highest on the 9th day, the CO_2_ efflux levels of biochar treatments were lower than those of CK, which was because the negative priming effect of biochar was strongest on the 9th day and was the dominant factor (Figure 4b). Moreover, the CO_2_ efflux of the BC1 and BC4 treatments decreased in the early stage but rose abruptly on the 9th day, because the CO_2_ efflux from biochar was highest on that day, but it should be noticed that the CK treatment also increased, indicating that other mechanisms may exist which are worthy of further study. Throughout the incubation period, the CO_2_ emissions of BC1 and BC4 were lower than those of CK, which was in line with the studies by Sakin et al. [16] and Herath et al. [13]. The ability of biochar addition to reduce soil CO_2_ emissions was attributed to the negative priming effect of biochar addition on native SOC mineralization [46,47]. Our results confirmed the negative priming effect (Figure 4b).

Our results are consistent with the results of the most recent studies, which indicate that the biochar addition inhibits N_2_O emission [17,18,19]. The mechanisms for inhibiting N_2_O emissions were as follows: (1) biochar contained a large number of soluble alkaline cations, which were released into the soil to increase the pH and then promote N_2_O reductase activity; more N_2_O was converted to N_2_, and this resulted in lower N_2_O emissions [48]. (2) Biochar addition enhanced soil aeration, and then reduced the abundance of denitrifying bacteria and inhibited denitrification, and reduced N_2_O emissions [49]. (3) Biochar reduced the NH_4_^+^ and NO_3_^−^ concentrations in the soil by physical and chemical adsorption, which reduced substrates for nitrification and denitrification, and reduced N_2_O emissions [50]. Except for physical and chemical adsorption, our study also found that biochar addition would reduce NH_4_^+^-N and NO_3_^−^-N by promoting their microbial assimilation. Thus, reduced substrates for nitrification and denitrification would explain the decrease in N_2_O emissions in our study. This mechanism needs to be verified by investigating the effect of biochar addition on the rates of NH_4_^+^-N bioassimilation and NO_3_^−^-N bioassimilation. The N primary transformation rates i.e., soil organic N mineralization rate, NH_4_^+^-N bioassimilation rate, NO_3_^−^-N bioassimilation rate, nitrification rate, ammonia volatilization rate and denitrification rate, can be measured by the ^15^N tracer technique [51]. Moreover, the contribution rates of various sources (organic N heterotrophic nitrification, NH_4_^+^-N autotrophic nitrification and denitrification) to soil N_2_O emissions can be quantitatively analyzed by the ^15^N tracer technique [51]. Determining the effect of the N primary transformation rates and the contribution rates of various sources provide foci for future research.

The CH_4_ emissions of BC1 were significantly lower than those of CK, which was consistent with most of the previous studies [23,24,25]. The reasons for the inhibition of CH_4_ emission by biochar addition were as follows: (1) Biochar improved soil aeration conditions, made the soil detrimental to the growth of methanogens and reduced CH_4_ production. Meanwhile, enhanced soil aeration could increase the oxidation of CH_4_, thereby reducing CH_4_ emissions [28]. (2) Soil pH was an important factor affecting CH_4_ production, and the size and structure of methanotrophic communities were more sensitive to the rising soil pH than those of methanogens, which is more beneficial to the methanotrophic communities, thus reducing CH_4_ emissions [52]. (3) Biochar can physically adsorb CH_4_, thus reducing CH_4_ emissions [53], and the CH_4_ absorbed by the biochar surface can be more effectively utilized by methanotrophs, which also contributes to reducing CH_4_ emissions [26]. It should be noted that the CH_4_ emissions of BC4 were higher than those of CK, but the difference was not significant. This may be because BC4 contained more unstable carbon from biochar, and provided more substrate for methanogens, promoting the production of CH_4_, which was supported by Feng et al. [23]. Investigating the relative contribution of CH_4_ production and oxidation provides insights into the effect mechanism of biochar on CH_4_ emissions. The relative contribution of CH_4_ production and oxidation can be quantified by the carbon stable isotope technique [54]. It is necessary to investigate the effect of biochar addition on the relative contribution of CH_4_ production and oxidation by using the carbon stable isotope technique in the future.

Our study found that biochar addition reduced GWP, but it did not decrease with increasing biochar addition ratios, which only partially supported the second hypothesis. The difference in GWP between BC1 and BC4 was not significant, because the significantly reduced N_2_O emissions of BC4 compared to BC1 were counteracted by the increased CH_4_ emissions in BC4. The emission patterns of CO_2_ were consistent with those of the GWP, and thus, CO_2_ emissions should be considered the main contributors to the GWP [55,56].

### 4.3. The Priming Effects of Biochar Addition on Soil Organic Carbon Mineralization

Our findings revealed that biochar addition had a negative priming effect on CO_2_ emissions, which is consistent with our third hypothesis. The negative priming effect may be attributed to: (1) the adsorption of SOC on the surface of biochar and the physical protection of SOC through the formation of microaggregates enhanced by biochar addition [57]; (2) the soluble organic carbon in biochar being preferentially used by microorganisms as a carbon source, thus protecting the native SOC [29]. The temporal pattern of the negative priming effect of biochar addition found in this study is consistent with Lu et al. [29], who also found the negative priming effect increased and then decreased with time, and gradually tended to a stable level in the later stage. This is due to the higher number of adsorption sites of biochar in the early stage, which enhances the physical protection of SOC by the formation of microaggregates. Meanwhile, biochar contains soluble organic carbon, which is a preferred carbon source and reduces the mineralization of native SOC. As a result, the negative priming effect is stronger in the early stage. With the extension of time, the adsorption sites were occupied and the soluble organic carbon in biochar was consumed, so the negative priming effect gradually decreased and stabilized. The above mechanism should be considered one of the mechanisms that made the negative priming effect of BC4 significantly stronger than that of BC1 in the early stage, though there was no significant difference in the late stage.

## 5. Conclusions

This study verified that cornstalk biochar addition can not only enhance soil physicochemical properties but also has the potential to mitigate climate change. Biochar addition reduced CO_2_ and N_2_O emissions, while BC1 significantly reduced CH_4_ emissions, and BC4 increased CH_4_ emissions. On the whole, biochar addition significantly reduced the GWP, but there was no significant difference between BC1 and BC4. The results suggest that 1% should be considered a more reasonable biochar addition ratio for mitigating greenhouse gas emissions in sandy loam and provide a theoretical basis for mitigating global warming by way of biochar addition. In this study, the negative priming effect of biochar addition on native SOC mineralization was verified by the ^13^C tracer technique, which was attributed to the physical protection of native SOC conducted by promoting microaggregate formation and preferentially using the soluble organic carbon in biochar. Our study also emphasizes that it is necessary to determine the effects of biochar addition on the N primary transformation rates and quantify the contribution rates of various nitrogen sources by the ^15^N tracer technique and quantify the relative contribution of CH_4_ production and oxidation by the carbon stable isotope technique in the future, which is important for comprehensively understanding the effect mechanisms of biochar addition on greenhouse gas emissions.

## Figures and Tables

**Table 1:** Physicochemical properties of soil and biochar.

	pH	CEC cmol kg^−1^	SOC g kg^−1^	TN g kg^−1^	NO_3_^−^-N mg kg^−1^	NH_4_^+^-N mg kg^−1^	δ^13^C ‰
Soil	7.19	10.9	14.59	0.69	24.67	0.30	−24.4
Biochar	8.81	13.25	601.42	21.39	-	-	−14.4

## Data Availability

The data presented in this study are available on request from the corresponding author.

## References

[1] IPCC (2018). Special Report on Global Warming of 1.5 °C.

[2] IPCC (2021). The Physical Science Basis. Contribution of Working Group I to the Sixth Assessment Report of the Intergovernmental Panel on Climate Change. https://www.ipcc.ch/report/ar6/wg1/#FullReport.

[3] Ibrahim S. (2016). Impact of greenhouse gases and climate change. Nature.

[4] IPCC (2014). Synthesis Report. Contribution of Working Groups I, II and III to the Fifth Assessment Report of the Intergovernmental Panel on Climate Change.

[5] Smith P. (2016). Soil carbon sequestration and biochar as negative emission technologies. Glob. Chang. Biol..

[6] Wang L., Gao C., Yang K., Sheng Y., Zhu L. (2021). Effects of biochar aging in the soil on its mechanical property and performance for soil CO_2_ and N_2_O emissions. Sci. Total Environ..

[7] Pokharel P., Kwak J.H., Ok Y.S., Chang S.X. (2018). Pine sawdust biochar reduces GHG emission by decreasing microbial and enzyme activities in forest and grassland soils in a laboratory experiment. Sci. Total Environ..

[8] Zhang Y., Wang J., Feng Y. (2021). The effects of biochar addition on soil physicochemical properties: A review. Catena.

[9] Mitchell P.J., Simpson A.J., Soong R., Simpson M.J. (2015). Shifts in microbial community and water-extractable organic matter composition with biochar amendment in a temperate forest soil. Soil Biol. Biochem..

[10] Tang Y., Gao W.C., Cai K., Chen Y., Li C.B., Lee X.Q., Cheng H.G., Zhang Q.H., Cheng J.Z. (2021). Effects of biochar amendment on soil carbon dioxide emission and carbon budget in the karst region of southwest China. Geoderma.

[11] Wang Q., Zeng Z., Zhong M. (2016). Soil Moisture Alters the Response of Soil Organic Carbon Mineralization to Litter Addition. Ecosystems.

[12] Yang X., Meng J., Lan Y., Chen W.F., Yang T.X., Yuan J., Liu S.N., Han J. (2017). Effects of maize stover and its biochar on soil CO_2_ emissions and labile organic carbon fractions in Northeast China. Agric. Ecosyst. Environ..

[13] Herath H., Camps-Arbestain M., Hedley M.J., Kirschbaum M., Wang T., Hale R.V. (2015). Experimental evidence for sequestering C with biochar by avoidance of CO_2_ emissions from original feedstock and protection of native soil organic matter. GCB Bioenergy.

[14] Li J.B., Kwak J.H., Chang S.X., Gong X., An Z., Chen J. (2021). Greenhouse gas emissions from forest soils reduced by Straw biochar and nitrapyrin applications. Land.

[15] Odugbenro G.O., Liu Z., Sun Y. (2019). Dynamics of C and N in a clay loam soil amended with biochar and corn straw. Indian J. Agric. Res..

[16] Sakin E., Ramazanoglu E., Seyrek A. (2021). Effects of Different Biochar Amendments on Soil Enzyme Activities and Carbondioxide Emission. Commun. Soil Sci. Plant Anal..

[17] Deng B.L., Zheng L.Y., Ma Y.C., Zhang L., Liu X.J., Zhang X.L., Zhang W.Y., Huang W., Hu X.F., Guo X.M. (2020). Effects of mixing biochar on soil N_2_O, CO_2_, and CH_4_ emissions after prescribed fire in alpine meadows of Wugong Mountain, China. J. Soils Sediments.

[18] Khan M.N., Li D.C., Shah A., Huang J., Zhang L., Núez-Delgado A., Han T.F., Du J.X., Ali S., Sial T.A. (2022). The impact of pristine and modified rice straw biochar on the emission of greenhouse gases from a red acidic soil. Environ. Res..

[19] Li H., Meng J., Liu Z.Q., Lan Y., Yang X., Huang Y.W., He T.Y., Chen W.F. (2021). Effects of biochar on N_2_O emission in denitrification pathway from paddy soil: A drying incubation study. Sci. Total Environ..

[20] Tang Z.M., Liu X.R., Li G.C., Liu X.W. (2022). Mechanism of biochar on nitrification and denitrification to N_2_O emissions based on isotope characteristic values. Environ. Res..

[21] Elizabeth V., Johan S. (2014). Biochar does not mitigate field-scale N_2_O emissions in a Northern California vineyard: An assessment across two years. Agric. Ecosyst. Environ..

[22] Lin Y., Ding W., Liu D., He T., Yoo G., Yuan J., Chen Z., Fan J. (2017). Wheat straw-derived biochar amendment stimulated N_2_O emissions from rice paddy soils by regulating the amoA genes of ammonia-oxidizing bacteria. Soil Biol. Biochem..

[23] Feng Y.Z., Xu Y.P., Yu Y.C., Xie Z.B., Lin X.G. (2012). Mechanisms of biochar decreasing methane emission from Chinese paddy soils. Soil Biol. Biochem..

[24] Qi L., Ma Z., Chang S.X., Zhou P., Gao M. (2020). Biochar decreases methanogenic archaea abundance and methane emissions in a flooded paddy soil. Sci. Total Environ..

[25] Wu Z., Zhang X., Dong Y.B., Xu X., Xiong Z.Q. (2018). Microbial explanations for field-aged biochar mitigating greenhouse gas emissions during a rice-growing season. Environ. Sci. Pollut. Res..

[26] Kubaczynski A., Walkiewicz A., Pytlak A., Galazka A., Brzezinska M., Grzadziel J. (2021). Biochar dose determines methane uptake and methanotroph abundance in Haplic Luvisol. Sci. Total Environ..

[27] Wu Y.Y., Hou P.F., Guo Z., Sun H.J., Li D.T., Xue L.H., Feng Y.F., Yu S., Yang L.Z., Xing B.S. (2021). Raw material of water-washed hydrochar was critical for the mitigation of GHGI in infertile paddy soil: A column experiment. Biochar.

[28] Brassard P., Godbout S., Raghavan V. (2016). Soil biochar amendment as a climate change mitigation tool: Key parameters and mechanisms involved. J. Environ. Manag..

[29] Lu W.W., Zhang H.L. (2015). Response of biochar induced carbon mineralization priming effects to additional nitrogen in a sandy loam soil. Appl. Soil Ecol..

[30] Bao S.D. (2008). Soil and Agro-Chemical Analysis.

[31] Zwieten L.V., Kimber S., Morris S., Downie A., Berger E., Rust J., Scheer C. (2010). Influence of biochars on flux of N_2_O and CO_2_ from Ferrosol. Soil Res..

[32] Aylward G.H., Finlay T.J.V. (1974). SI Chemical Data.

[33] Beetz S., Liebersbach H., Glatzel S., Jurasinski G., Buczko U., Hoeper H. (2013). Effects of land use intensity on the full greenhouse gas balance in an Atlantic peat bog. Bio. Geosci..

[34] Lu W.W., Ding W.X., Zhang J.H., Li Y., Luo J.F., Bolan N., Xie Z.B. (2014). Biochar suppressed the decomposition of organic carbon in a cultivated sandy loam soil: A negative priming effect. Soil Biol. Biochem..

[35] Pataki D.E., Ehleringer J.R., Flanagan L.B., Yakir D., Bowling D.R., Still C.J., Buchmann N., Kaplan J.O., Berry J.A. (2003). The application and interpretation of Keeling plots in terrestrial carbon cycle research. Glob. Biogeochem. Cycles.

[36] Stocker T.F., Qin D., Plattner G.-K., Tignor M., Allen S., Boschung J., Nauels A., Xia Y., Bex V., Midgley P. (2014). Climate Change 2013: The Physical Science Basis: Working Group I Contribution to the Fifth Assessment Report of the Intergovernmental Panel on Climate Change.

[37] Gan W.J., He Y., Zhang X.F., Zhang S.T., Lin Y.S. (2012). Effects and Mechanisms of Straw Biochar on Remediation Contaminated Soil in Electroplating Factory. J. Ecol. Rural Environ..

[38] Jones D.L., Murphy D.V., Khalid M., Ahmad W., Edwards-Jones G., Deluca T.H. (2011). Short-term biochar-induced increase in soil CO_2_ release is both biotically and abiotically mediated. Soil Biol. Biochem..

[39] Jien S.H., Wang C.S. (2013). Effects of biochar on soil properties and erosion potential in a highly weathered soil. Catena.

[40] Kharel G., Sacko O., Feng X., Morris J.R., Phillips C.L., Trippe K., Kumar S., Lee J.W. (2019). Biochar Surface Oxygenation by Ozonization for Super High Cation Exchange Capacity. ACS Sustain. Chem. Eng..

[41] Maestrini B., Nannipieri P., Abiven S. (2015). A meta-analysis on pyrogenic organic matter induced priming effect. GCB Bioenergy.

[42] Murtaza G., Ahmed Z., Usman M., Tariq W., Ullah Z., Shareef M., Iqbal H., Waqas M., Tariq A., Wu Y.F. (2021). Biochar induced modifications in soil properties and its impacts on crop growth and production. J. Plant Nutr..

[43] Janssen B.H. (1996). Nitrogen mineralization in relation to C:N ratio and decomposability of organic materials. Plant Soil.

[44] Nishio T., Komada M., Arao T., Kanamori T. (2001). Simultaneous determination of transformation rates of nitrate in soil. Jpn. Agric. Res. Q..

[45] Wang J., Xiong Z., Kuzyakov Y. (2016). Biochar stability in soil: Meta-analysis of decomposition and priming effects. GCB Bioenergy.

[46] Kerre B., Hernandez-Soriano M.C., Smolders E. (2016). Partitioning of carbon sources among functional pools to investigate short-term priming effects of biochar in soil: A ^13^C study. Sci. Total Environ..

[47] Zheng T., Zhang J., Tang C., Liao K., Guo L. (2021). Positive and negative priming effects in an Ultisol in relation to aggregate size class and biochar level. Soil Tillage Res..

[48] Dong W., Walkiewicz A., Bieganowski A., Oenema O., Nosalewicz M., He C., Zhang Y., Hu C. (2020). Biochar promotes the reduction of N_2_O to N_2_ and concurrently suppresses the production of N_2_O in calcareous soil. Geoderma.

[49] Shi Y., Liu X., Zhang Q., Li Y. (2022). Contrasting effects of biochar and organic fertilizer-amendment on community compositions of nitrifiers and denitrifiers in a wheat-maize rotation system. Appl. Soil Ecol..

[50] Xu X., Yuan X., Zhang Q., Wei Q., Liu X., Deng W., Wang J. (2021). Biochar derived from spent mushroom substrate reduced N_2_O emissions with lower water content but increased CH_4_ emissions under flooded condition from fertilized soils in Camellia oleifera plantations. Chemosphere.

[51] Liao X., Muller C., Jansen-Willems A., Luo J.F., Lindsey S., Liu D.Y., Chen Z.M., Niu Y.H., Ding W.X. (2021). Field-aged biochar decreased N_2_O emissions by reducing autotrophic nitrification in a sandy loam soil. Biol. Fertil. Soils.

[52] Jeffery S., Verheijen F.G.A., Kammann C., Abalos D. (2016). Biochar effects on methane emissions from soils: A meta-analysis. Soil Biol. Biochem..

[53] Sadasivam B.Y., Reddy K.R. (2015). Adsorption and transport of methane in landfill cover soil amended with waste-wood biochars. J. Environ. Manag..

[54] Zhang G.B., Zhang W.X., Yu H.Y., Ma J., Xu H., Yagi K. (2015). Fraction of CH_4_ oxidized in paddy field measured by stable carbon isotopes. Plant Soil.

[55] Cui Y., Li N., Chen L. (2021). Carbon neutrality and mitigating contribution of terrestrial carbon sink on anthropogenic climate warming in China, the United States, Russia and Canada. J. Geogr. Sci..

[56] Li N., Cui Y.P., Fu Y.M., Liu X.Y., Run Y.D., Li M.D., Chen L.Y., Xia H.M., Lu H.L. (2021). Contribution of anthropogenic CO_2_ in China to global radiative forcing and its offset by the ecosystem during 2000–2015. Ann. N. Y. Acad. Sci..

[57] Gross C.D., Bork E.W., Carlyle C.N., Chang S.X. (2022). Biochar and its manure-based feedstock have divergent effects on soil organic carbon and greenhouse gas emissions in croplands. Sci. Total Environ..

